# Epigenetic plasticity and chemoresistance in cancer: mechanisms, biomarkers, and translational opportunities for real-world evidence

**DOI:** 10.3389/fcell.2026.1786398

**Published:** 2026-06-18

**Authors:** Cadiele Oliana Reichert, Nélio Cézar de Aquino, Vinícius de Camargo Callefi, Isadora Alves, Sofia Cattena, Emanuelle Rocha Santos, Ketelyn Aparecida Deamo Vasconcelos, Hebert Fabricio Culler, Luis Alberto de Pádua Covas Lage, Vanderson Rocha, Adriana Castello Costa Girardi, Carlos Alejandro Murga-Zamalloa, Juliana Pereira

**Affiliations:** 1 Laboratory of Medical Investigation in Pathogenesis and Directed Therapy in Onco-Immuno-Hematology (LIM-31), Department of Hematology, Hemotherapy and Cell Therapy - Faculty of Medicine, University of São Paulo (FM-USP), SãoPaulo, Brazil; 2 Real-World Evidence Observatory and Lab for Precision Public Health, Department of Hematology, Hemotherapy and Cell Therapy - Faculty of Medicine, University of São Paulo (FM-USP), SãoPaulo, Brazil; 3 Brazilian Health Regulatory Agency (Agência Nacional de Vigilância Sanitária - ANVISA), Brasília, Brazil; 4 Cancer Institute of The State of São Paulo (ICESP) Octávio Frias de Oliveira, SãoPaulo, Brazil; 5 Laboratory of Renal Physiology and Cardiometabolism, Department of Cardiopneumology, University of São Paulo (FMUSP), SãoPaulo, Brazil; 6 Department of Pathology, University of Illinois at Chicago, Chicago, IL, United States

**Keywords:** chemoresistance, DNA methylation, epigenetic therapy, epigenomics, real-world evidence

## Abstract

Chemoresistance remains a major barrier to durable cancer control and is increasingly understood as a dynamic process shaped not only by genetic selection, but also by epigenetically regulated changes in cellular state. Evidence supports a model in which a subset of tumor cells survives treatment through drug-tolerant persister states characterized by slow cycling, stress tolerance, transcriptional rewiring, and altered interactions with the tumor microenvironment. In this context, chromatin remodeling, histone-state regulation, chromatin accessibility, enhancer reprogramming, and lineage plasticity emerge as central mechanisms enabling adaptive survival under therapeutic pressure and facilitating transition toward more stable resistant phenotypes. These mechanisms also provide a biological rationale for epigenetic therapies as priming, combination, or resensitization strategies. Clinical and translational studies suggest that targeting epigenetic regulators may help restore treatment susceptibility, although current evidence remains heterogeneous, with variable regimens, endpoints, and biomarker sampling strategies. Epigenomic biomarkers may therefore be particularly valuable for identifying adaptive cell states, monitoring target engagement, and tracking resistant trajectories over time. Real-world data and real-world evidence can complement mechanistic and clinical studies by capturing treatment sequencing, heterogeneous populations, and post-approval effectiveness and safety patterns. However, their translational value depends on fit-for-purpose design, analytical validity, transparent provenance, and bias-aware methods, particularly in sequential treatment settings prone to confounding, endpoint misclassification, and non-random molecular testing. In this mini-review, we examine how epigenetic plasticity drives chemoresistance and how epigenomic biomarkers and real-world data may support clinical research and more rigorous evidence generation.

## 1 Introduction

Chemoresistance is increasingly understood as a dynamic process shaped by both genomic evolution and epigenetic plasticity (Levy et al., 2020; Pradhan et al., 2026; Lage et al., 2023; Alexandrova et al., 2022; de Pádua Co et al., 2022). Although genetic mutations confer long-term resistance, the transient survival of drug-tolerant persisters (DTPs) represents a vital non-genetic precursor (Russo et al., 2024; Sharma et al., 2010). These cells leverage metabolic and transcriptional rewiring to navigate initial therapeutic stress, eventually transitioning into stable refractory states. Thus, chemoresistance can be more accurately viewed as the product of both clonal selection and therapy-induced phenotypic plasticity (Ramirez et al., 2016; Seidel and von Karstedt, 2022; Zhang and Goodrich, 2022; Li et al., 2026).

Within this model, resistance may initially emerge through epigenetic adaptation rather than immediate genomic escape. By enabling rapid chromatin remodeling and transcriptional reprogramming without DNA sequence alterations, epigenetic plasticity drives processes such as enhanced DNA repair, apoptotic evasion, metabolic rewiring, and the acquisition of stem-like properties or lineage instability. These mechanisms provide a strong rationale for the development of epigenetic therapeutics and epigenomic biomarkers. However, their translational utility depends on analytically validated assays, reproducible protocols, and clear methodological and translational standards (Alexandrova et al., 2022; Matei et al., 2012; Wang N. et al., 2023; Li et al., 2021; Chen et al., 2024; Malone and Roberts, 2024; Song et al., 2025; Soragni et al., 2025; Tao et al., 2024).

Real-world data (RWD) and real-world evidence (RWE) can extend the evaluation of therapeutic effectiveness beyond conventional trials by capturing longitudinal treatment trajectories, heterogeneous populations, and post-approval clinical outcomes (Sherman et al., 2016; Purpura et al., 2022; FDA, 2018; Lau et al., 2022). This is especially relevant in chemoresistance, where clinical benefit is often distributed across sequential treatment decisions rather than a single uniform endpoint. However, these settings magnify threats to causal inference, including confounding by indication, time-zero misalignment, and informative missingness. Accordingly, RWD/RWE are most informative when they are fit for purpose, transparently designed, and analytically aligned with the clinical estimand of interest (Timbie et al., 2021; D et al., 2022; Hiramatsu et al., 2021; Krause and Saver, 2018; Burns et al., 2022; Dang, 2023; Tan et al., 2022; Gatto et al., 2023; Lord et al., 2025).

**FIGURE 1:**
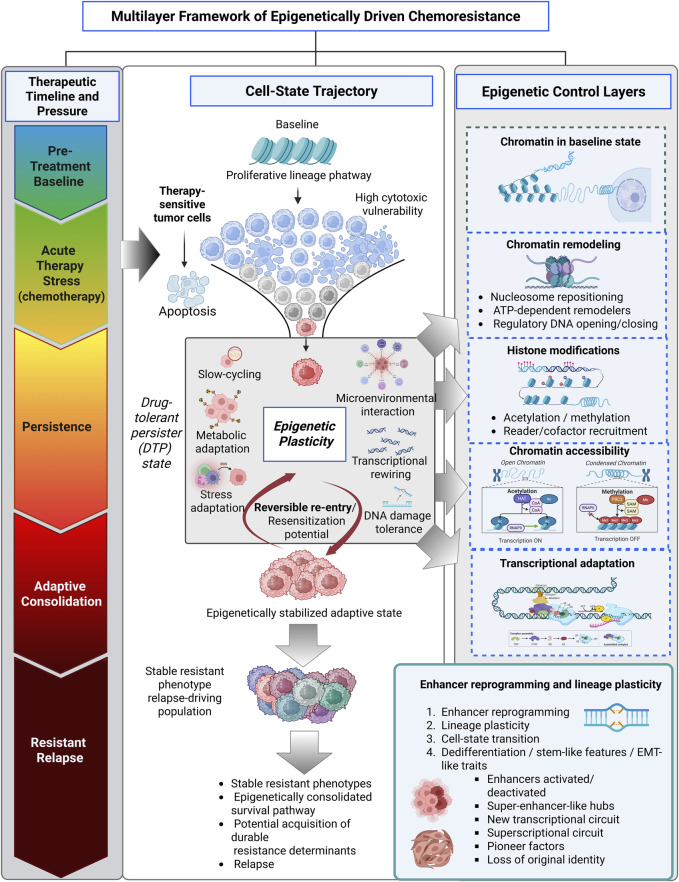
Epigenetic plasticity drives DTP formation and resistant cell-state transitions. Reversible DTP states are sustained by chromatin and transcriptional rewiring. Progressive epigenetic adaptation, including enhancer reprogramming and lineage plasticity, drives the transition from transient survival to stable treatment-refractory phenotypes.

In this mini-review, we first examine the mechanistic basis of epigenetic plasticity in chemoresistance, focusing on DTP states, chromatin remodeling, and enhancer reprogramming. Figure 1 summarizes the mechanistic basis discussed in the following sections. We then discuss how epigenomic biomarkers and RWD can support translational evidence generation when anchored in robust biology and methodologically disciplined study design.

## 2 Epigenetic plasticity and drug-tolerant persister states in chemoresistance

Chemoresistance is increasingly understood as a dynamic and multistep process that extends beyond the gradual selection of fixed genetic alterations. A substantial fraction of tumor cells can survive treatment through reversible, non-mutational cell-state transitions (Shaffer et al., 2017). These transient survival states, commonly termed DTP states, are characterized by reduced proliferative output, stress adaptation, broad transcriptional rewiring, and altered interactions with the tumor microenvironment (Russo et al., 2024; Sharma et al., 2010; Hangauer et al., 2017; Pu et al., 2023). Rather than representing a terminal phenotype, DTPs are better viewed as an intermediate adaptive compartment that enables survival under acute therapeutic pressure and may subsequently seed more stable resistant phenotypes (Wang et al., 2025; Liu S. et al., 2025; Joun et al., 2025; Lalla et al., 2026; Ghoderao et al., 2025).

This view recasts chemoresistance as a continuum of therapy-induced cell-state transitions rather than a static binary outcome. Early resistance need not arise from an immediately fixed genomic escape mechanism. Tumor cells may initially survive by entering a stress-tolerant state marked by reduced proliferation, destabilized lineage pathways, and reorganized survival pathways (Musella et al., 2022; Galluzzi and Kroemer, 2022). The biological relevance of this concept was established by the demonstration that a reversible drug-tolerant state can be chromatin-mediated and therapeutically targetable, placing epigenetic regulation at the center of early adaptation to treatment (Russo et al., 2024; Sharma et al., 2010).

A central implication of the DTP model is that epigenetic plasticity provides the regulatory architecture through which these adaptive states are generated and maintained. Because chromatin states can be remodeled rapidly, epigenetic regulation can alter transcriptional output without requiring stable DNA-sequence change. This enables tumor cells to adopt phenotypes optimized for survival under chemotherapeutic stress through coordinated changes in chromatin accessibility, histone-associated regulatory states, enhancer activity, and transcriptional circuitry. Epigenetic plasticity should therefore be viewed not merely as an accompanying feature of resistance, but as an enabling mechanism of adaptive survival (Wang N. et al., 2023; Flavahan et al., 1979; Strauss and Figg, 2015; Zhu et al., 2023; Dawson and Kouzarides, 2012).

Chemotherapy imposes profound selective and physiological stress. To persist, tumor cells must suppress cell death pathways, tolerate replicative and metabolic stress, preserve DNA damage handling capacity, and often loosen their pre-existing differentiation identity. DTPs should therefore not be regarded as passive residual cells, but as actively maintained adaptive states in which cancer cells move away from proliferation and toward persistence. Their reversible nature suggests that early resistance may remain biologically unstable, and therefore potentially tractable, before more entrenched resistant phenotypes emerge. This view also links short-term persistence with longer-term disease evolution, because cells that survive in a reversible tolerant state may later undergo phenotypic consolidation or acquire more stable resistance determinants (Bogan and Yi, 2024; Eckersley-Maslin, 2020).

Epigenetic plasticity places cell-state regulation at the center of chemoresistance under therapeutic stress. Within this model, DTP cells occupy a pivotal position by demonstrating how tumor cells survive treatment through reversible, chromatin-mediated adaptation (Russo et al., 2024; Sharma et al., 2010; Soragni et al., 2025).

## 3 Chromatin remodeling, histone modifications, and transcriptional adaptation to therapy

Adaptive chemoresistance is characterized by rapid gene expression reorganization before stable genetic resistance is established. This process depends on dynamic chromatin regulation, which dictates the accessibility of regulatory DNA to transcription factors and co-regulators. In this setting, chromatin remodeling, changes in chromatin accessibility, and histone-state control act as major determinants of whether a cell leaves a therapy-sensitive state and enters one permissive for survival (Malone and Roberts, 2024; França et al., 2024).

Chromatin remodeling provides the structural basis for this adaptability. ATP-dependent complexes regulate nucleosome positioning and spacing to control access to promoters, enhancers, and other cis-regulatory elements. In cancer, these complexes are critical because their alteration influences lineage fidelity and the capacity to exploit alternative regulatory programs under stress. During therapy, remodeling facilitates the transition to transcriptional states compatible with persistence rather than drug sensitivity. In parallel, changes in chromatin accessibility selectively activate new transcription factor networks while restricting prior lineage-defining circuits. Such accessibility changes are likely to contribute directly to resistance-associated state transitions rather than merely reflect them (Sharma et al., 2010; Dawson and Kouzarides, 2012; Sundaram et al., 1979).

A second, functionally coupled layer is provided by histone modifications. Cancer-associated histone modifications are primarily represented by acetylation, methylation, phosphorylation, and ubiquitination, although emerging evidence also implicates lactylation, SUMOylation, and other noncanonical marks (Gatla et al., 2017; Duan et al., 2024; Kawaf et al., 2024; Liu F. et al., 2025). Histone acetylation, controlled by HATs/KATs and HDACs, is generally associated with transcriptional activation through chromatin opening. In cancer, global loss of H4K16 monoacetylation is an early and progressive hallmark of tumorigenesis, while promoter hypoacetylation contributes to silencing of tumor suppressor genes even in the absence of DNA methylation (Gatla et al., 2017; Duan et al., 2024; Kawaf et al., 2024; Liu F. et al., 2025).

Conversely, aberrant activity of acetyltransferases such as p300/CBP and KAT6A/B may sustain oncogenic transcription. Histone methylation has context-dependent effects determined by the modified residue and methylation state. Oncogenic gain-of-function mutations in EZH2, particularly at Y641 in follicular lymphoma, increase H3K27me3 and promote repression of tumor suppressor genes. Additional methyltransferases involved in cancer include NSD2, SETD2, and DOT1L (Ramirez et al., 2016). More broadly, cancers frequently exhibit loss of activating marks such as H3K4me2/me3 and gain of repressive marks including H3K9me2/me3 and H3K27me3 (Alexandrova et al., 2022; Gatla et al., 2017; Duan et al., 2024; Kawaf et al., 2024; Liu F. et al., 2025; Al et al., 2018).

In therapy-exposed cells, these regulatory states may reinforce survival-promoting programs while dampening transcription linked to proliferation and differentiation. This is especially relevant in slow-cycling, stress-tolerant phenotypes, in which tumor cells must rebalance DNA damage handling, apoptosis control, oxidative stress responses, and metabolic adaptation. Histone-state regulation therefore stabilizes broader adaptive phenotypes, including stemness-like and immune-evasive states (Al et al., 2018; Guo et al., 2022; Moore et al., 2023; Palma et al., 2024).

Together, chromatin remodeling, changes in chromatin accessibility, and histone-state control converge on transcriptional adaptation. Under chemotherapy, tumor cells experience acute pressure on replication, proteostasis, metabolism, and DNA repair. Survival requires reprioritization of transcription away from the original growth program and toward state-specific survival modules. These programs commonly converge on stress tolerance, apoptotic buffering, repair capacity, metabolic flexibility, and partial destabilization of lineage identity. This model also supports epigenetic therapies as rational resensitization or combination strategies, particularly when directed against adaptive chromatin states before resistant phenotypes become fixed. Mechanistically, epigenetically mediated resistance appears to be driven mainly by reversible cell-state entry, changes in chromatin accessibility, enhancer redistribution, and progressive loss of lineage constraint (Matei et al., 2012; Soragni et al., 2025; França et al., 2024; Al et al., 2018; Guo et al., 2022; Moore et al., 2023; Palma et al., 2024; Teneng et al., 2019).

## 4 Enhancer reprogramming, lineage plasticity, and resistant cell-state transitions

If chromatin remodeling and histone-state regulation provide the permissive architecture for adaptive resistance, enhancer reprogramming helps specify the transcriptional identity adopted by persisting or resistant cells. Enhancers are context-dependent regulatory elements that integrate transcription factors, chromatin accessibility, signaling inputs, and co-regulator recruitment to control lineage-restricted gene expression (Fagnocchi et al., 2018; Zboril et al., 2021). Under therapeutic pressure, enhancer landscapes can be reconfigured, allowing malignant cells to activate alternative transcriptional circuits, attenuate lineage-constraining programs, and stabilize survival in drug-exposed states. Enhancer reprogramming can therefore be viewed as a core mechanism through which epigenetic plasticity is translated into durable phenotypic change (Perlman et al., 2024; Mehta and Stanger, 2024).

This process is especially relevant when the pre-treatment transcriptional state is no longer compatible with survival. In that setting, tumor cells can redistribute enhancer activity toward programs that support stress tolerance, reduced drug dependence, and altered cell identity. By reconfiguring the transcription factor networks that dominate enhancer usage, cancer cells can move away from a therapy-sensitive lineage state and toward adaptive phenotypes better suited to persistence. Recent work in prostate cancer illustrates this principle, showing that FOXA2-dependent rewiring of AP-1-centered circuitry can drive lineage plasticity through large-scale transcriptional reprogramming (Wang et al., 2024).

A useful distinction can be made between state adaptation and lineage re-specification (França et al., 2024; Zippo and Beyes, 2025). In some tumors, therapy induces a tolerant state in which cells remain broadly related to the original lineage but acquire sufficient regulatory flexibility to persist (Barkley et al., 2021). In others, prolonged selective pressure favors high-lineage plasticity, with cells losing features of their prior differentiated program and adopting an alternative identity that is intrinsically less sensitive to treatment (Chan et al., 2026). In prostate cancer, LKB1 inactivation has been shown to promote epigenetic remodeling, DNA hypomethylation, and AR-independent lineage plasticity linked to antiandrogen resistance (Liang et al., 2025; Hermanova et al., 2020). More broadly, lineage plasticity is now framed as a major organizing principle of therapeutic escape (Liu S. et al., 2025; Mehta and Stanger, 2024; Li et al., 2025).

Enhancer reprogramming also helps explain why resistant states frequently display stem-like features, dedifferentiation, or partial developmental regression. Once an enhancer-rewired state is established, tumor cells may acquire new transcriptional dependencies rather than becoming uniformly diffuse or untargetable. These dependencies can reside in super-enhancer circuits, pioneer factor partnerships, higher-order enhancer-promoter assemblies, or lineage-specific co-activator networks (Song et al., 2025; Tao et al., 2024; Zhu et al., 2023). Topological alterations in enhancer-promoter hubs have been shown to coincide with transcriptional changes associated with acquired resistance, supporting the idea that resistant identity can be stabilized at the level of regulatory architecture itself (Perlman et al., 2024).

Recent single-cell chromatin accessibility atlases across human cancers underscore the feasibility of resolving malignant regulatory states at high resolution, supporting epigenomic approaches as tools for tracking adaptive trajectories rather than only late resistant endpoints (Sundaram et al., 1979). These transitions are also biomarker-relevant. If tumors evade therapy by changing identity rather than only by mutating a target, endpoint-based biomarkers may miss the process in progress. More informative readouts may capture enhancer usage, chromatin accessibility, and lineage transcriptional circuitry.

Taken together, these mechanisms are better viewed as linked components of a resistance continuum rather than as isolated events. DTP formation reflects an early reversible survival state under therapeutic stress; chromatin remodeling and histone-state regulation provide the permissive and stabilizing architecture for transcriptional adaptation; enhancer reprogramming helps specify the resistant transcriptional identity; and lineage plasticity represents a more advanced stage in which adaptive survival may consolidate into durable therapeutic escape. In this model, resistance is driven less by isolated epigenetic alterations than by coordinated changes in chromatin accessibility, regulatory circuitry, and cell identity over time.

## 5 Epigenomic biomarkers and real-world data in translational chemoresistance research

The mechanistic model outlined above has clear translational implications. If chemoresistance is driven, at least in part, by epigenetic plasticity, drug-tolerant persister states, chromatin remodeling, and lineage reprogramming, then epigenomic biomarkers can do more than describe molecular profiles. They may help define cell state, assess target engagement, estimate treatment susceptibility, and track adaptive rewiring over time. In this context, biomarkers may help identify tumors most likely to benefit from epigenetic priming, confirm on-target chromatin modulation, detect early exit from tolerant states, and monitor compensatory chromatin rewiring or lineage switching during treatment (ICH. Definitions for Genomic Biomarkers, 2007; Eustermann et al., 2024; Klemm et al., 2019).

This issue is especially relevant because the current evidence for epigenetic resensitization remains promising but methodologically heterogeneous. Small nonrandomized cohorts, variable priming regimens, endpoint ambiguity, limited serial biomarker sampling, and informative missingness weaken causal interpretation. The platinum-resistant ovarian cancer study combining low-dose decitabine with carboplatin illustrates biological plausibility (Matei et al., 2012) and broader reviews support targeting epigenetic regulators to overcome therapeutic resistance (Song et al., 2025).

Clinical examples help illustrate how epigenetic mechanisms may manifest as observable treatment patterns rather than remain purely conceptual. In platinum-resistant ovarian cancer, low-dose decitabine administered before carboplatin provided one of the clearest early examples of epigenetic resensitization, with biologic and clinical activity consistent with reversal of methylation-associated platinum resistance (Matei et al., 2012). By contrast, a randomized phase II study in partially platinum-sensitive ovarian cancer showed that decitabine plus carboplatin, on that specific schedule, reduced treatment deliverability and did not improve efficacy, underscoring that the clinical value of epigenetic priming depends on disease context, dosing, and patient selection (Glasspool et al., 2014).

In refractory non-small cell lung cancer, combined azacitidine and entinostat produced objective durable responses and was also linked to a blood-based biomarker associated with benefit (Juergens et al., 2011), while in advanced breast cancer the same combination was tested on the rationale that epigenetic therapy might restore therapeutically relevant transcriptional programs such as estrogen receptor expression and thereby support subsequent endocrine re-challenge (Connolly et al., 2017). Collectively, these studies suggest that DNA methylation and histone-state modulation are not only biologically plausible contributors to adaptive resistance, but may also shape clinically detectable patterns of resensitization, heterogeneous benefit, and context-dependent failure (Glasspool et al., 2014; Juergens et al., 2011; Connolly et al., 2017).

Additional examples illustrate how specific epigenetic states may serve as clinically informative biomarkers of treatment sensitivity or resistance. The classic case is glioblastoma, in which MGMT promoter methylation identifies tumors more likely to benefit from temozolomide, directly linking an epigenetic silencing event to chemotherapy response (Hegi et al., 2005). In high-grade serous ovarian cancer, quantitative BRCA1 promoter methylation has also shown translational relevance: homozygous or hemizygous BRCA1 methylation predicted rucaparib response in translational and clinical analyses, whereas methylation loss after chemotherapy was associated with loss of benefit, supporting the idea that epigenetic reversion can accompany acquired resistance (Kondrashova et al., 2018).

Consistent with this, liquid-biopsy studies have shown that BRCA1 promoter hypermethylation in circulating tumor DNA is often reversed at recurrence, a pattern interpreted as selection of therapy-resistant clones and linked to less favorable clinical trajectories (Elaze et al., 2021). These examples are especially relevant because they illustrate that epigenetic biomarkers may capture not only baseline susceptibility, but also dynamic escape from initially targetable states (Hegi et al., 2005; Kondrashova et al., 2018; Elaze et al., 2021).

Emerging plasma epigenomic approaches further strengthen the link between mechanism and longitudinal clinical observation by making resistant cell-state transitions measurable over time. In advanced prostate cancer, a targeted plasma DNA methylation assay was able to identify neuroendocrine prostate cancer non-invasively, supporting patient stratification in a treatment-resistant lineage-plastic state that is otherwise difficult to document serially (Franceschini et al., 2024). Complementing this, plasma cell-free DNA chromatin immunoprecipitation profiling of enhancer- and promoter-associated histone marks captured phenotypic and clinical heterogeneity in advanced prostate cancer, supporting chromatin-based liquid biopsy as a readout of adaptive tumor state (Sipola et al., 2025).

Most directly, serial plasma epigenomic profiling has detected treatment-emergent squamous transformation in prostate cancer, showing that lineage switching and transcriptional rewiring can be followed in real time rather than inferred only retrospectively from tissue relapse (Semaan et al., 2025). In a real-world or longitudinal care setting, these are the kinds of observations that could make epigenetic plasticity clinically actionable: not simply as a *post hoc* explanation of resistance, but as a dynamic process that can be tracked across sequential treatments, repeat sampling, and evolving disease states (Franceschini et al., 2024; Sipola et al., 2025; Semaan et al., 2025; Xiang et al., 2025).

However, stronger inference still requires either randomized evidence, careful quasi-experimental RWD analyses, or triangulation between mechanistic epigenomic findings and clinical data interpreted with explicit attention to bias. This also illustrates how molecular and clinical real-world data can be integrated within a structured translational approach that supports interoperability, data quality, and ethical use (Benchimol et al., 2015; Wang SV. et al., 2023; Huang et al., 2025; Nianogo et al., 2023; Desai et al., 2026; Cashin et al., 2025). This is particularly relevant in chemoresistance, where treatment is sequential and benefit is often reflected by later-line effectiveness, prolonged time-to-next-treatment, or delayed progression rather than by a single binary endpoint (Hernán and Robins, 2016).

Integrating epigenomics with RWD is most effective when tied to specific clinical objectives and fit-for-purpose endpoints (Cézar de Aquino et al., 2026). In single-arm settings, epigenomic data may improve comparability by providing molecularly coherent eligibility and pharmacodynamic anchoring, although it cannot resolve confounding by indication, time-zero misalignment, or baseline data gaps (ICH. Definitions for Genomic Biomarkers, 2007; FDA, 2023a; EMA, 2021; ICH, 2000). In post-approval settings, linked systems can extend evaluation to broader populations and inform longer-term therapeutic use (ICH, 2005; ICH, 2024; ENCePP, 2023; EMA, 2025). For clinical translation, biomarker development and companion diagnostic strategies require a defined context of use, structured pathways, and reproducible reporting standards (ICH. Definitions for Genomic Biomarkers, 2007; Benchimol et al., 2015; Wang SV. et al., 2023; FDA, 2011; Wang et al., 2021). Finally, RWD may help refine treatment sequencing, including questions of priming duration and regimen selection, although such analyses remain vulnerable to time-dependent confounding, immortal time bias, and informative censoring (Hernán and Robins, 2016; ENCePP, 2023; Wallach et al., 2023; Anvisa, 2023; FDA, 2024).

The main barriers to translational use in integrated epigenomics-RWD studies are often methodological and operational rather than biological (Berdasco and Esteller, 2019; Athieniti and Spyrou, 2023; Cazaly et al., 2019). Key sources of fragility include incomplete data provenance, poor linkage between omics results and source clinical records, assay instability, endpoint inconsistency, non-random molecular testing, and missing-not-at-random clinical or tissue data. For this reason, a concise methodological and translational perspective still adds value, but it should remain secondary to the biological focus of the review (ICH. Definitions for Genomic Biomarkers, 2007; FDA, 2023a; EMA, 2021; ICH, 2000; ICH, 2005; ICH, 2024; EMA, 2025; FDA, 2011; Anvisa, 2023; FDA, 2024; FDA, 2023b; FDA, 2023c; ICH, 2019; IC H, 2021; ICH, 2025). Overall, epigenomic biomarkers and RWD should be framed not as substitutes for mechanistic or prospective clinical studies, but as complementary translational tools.

**FIGURE 2:**
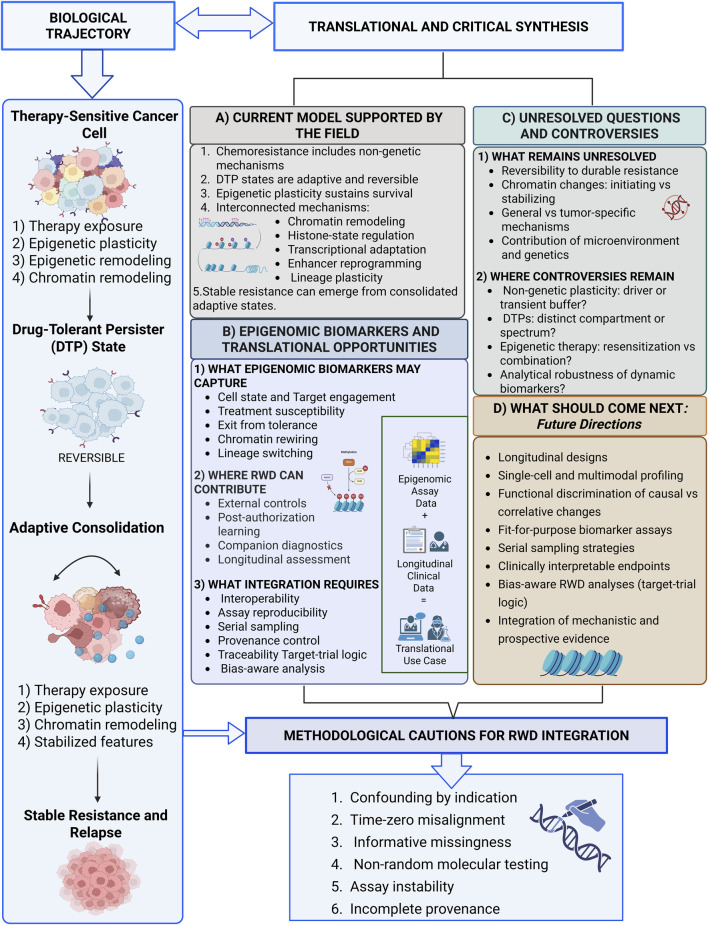
Integrated conceptual model linking mechanistic adaptation to translational application in chemoresistance. The upper panel summarizes the transition from therapy-sensitive cells to DTP states, adaptive consolidation, and stable resistance/relapse. The lower panel highlights four domains addressed in this review: current mechanistic understanding, translational opportunities for epigenomic biomarkers and RWD, unresolved questions and controversies, and future priorities. The bottom bar summarizes major methodological cautions for RWD integration.

As summarized in Figure 2, epigenetic adaptation in chemoresistance can be linked to biomarker development, real-world evidence generation, unresolved questions, and future research priorities within a single translational context.

## 6 Discussion

### 6.1 What the field now supports

Current evidence supports a model in which chemoresistance is driven not only by genetic selection, but also by epigenetically regulated cell-state adaptation. Within this model, DTP states, chromatin remodeling, histone-state regulation, transcriptional adaptation, enhancer reprogramming, and lineage plasticity can be understood not as separate mechanisms, but as interdependent layers of a resistance continuum that enables tumor survival under therapeutic pressure and, over time, stabilizes escape from treatment. This model has moved the field beyond a purely endpoint-based view of resistance and toward a dynamic view in which tumor cells can survive treatment through reversible and adaptive phenotypic transitions before, or in parallel with, the establishment of more stable resistant states (Russo et al., 2024; Sharma et al., 2010; Song et al., 2025; Soragni et al., 2025; Wang et al., 2025).

A second major advance is the recognition that these adaptive states are not only biologically meaningful, but also potentially actionable therapeutically. If early resistance is sustained by chromatin-mediated plasticity, then epigenetic therapies may be most effective when deployed to disrupt adaptive states before resistant phenotypes become fully entrenched. This model also supports the development of epigenomic biomarkers that capture state transitions, target engagement, and adaptive rewiring over time rather than relying only on static endpoint classification.

### 6.2 What remains unresolved

Despite this conceptual progress, several major questions remain unresolved. The first is mechanistic: it is still unclear how reversible persister states transition into more durable resistant phenotypes, and whether this progression is primarily driven by progressive chromatin reinforcement, enhancer rewiring, lineage re-specification, microenvironmental selection, or interaction with subsequent genetic evolution (Goyal et al., 2023; Ciriello et al., 2024). The second is temporal: most available studies do not adequately resolve when adaptive chromatin changes are initiating events, when they are stabilizing events, and when they are merely secondary correlates of persistence. The third is contextual: many of the mechanisms discussed here, including chromatin remodeling, enhancer switching, stemness-associated programs, and lineage plasticity, appear to be highly tumor-type- and treatment-dependent, which complicates the distinction between general principles and lineage-specific trajectories (Malone and Roberts, 2024; Mehta and Stanger, 2024; Li et al., 2025).

There are also major translational limitations. The clinical literature supporting epigenetic resensitization remains heterogeneous, with substantial variation in priming regimens, endpoints, sampling strategies, and molecular readouts. Serial biomarker collection is often sparse. Assay reproducibility and cross-platform comparability are not consistently demonstrated. In parallel, real-world evidence remains vulnerable to confounding by indication, time-zero misalignment, endpoint inconsistency, non-random molecular testing, and informative missingness. These issues limit causal interpretation and complicate efforts to connect mechanistic plausibility with clinically credible evidence (Benchimol et al., 2015; Hernán and Robins, 2016; Wang et al., 2021; Wallach et al., 2023).

### 6.3 Where the controversies remain

Several controversies continue to shape the field. One concerns causality: whether non-genetic plasticity is a primary driver of therapeutic escape or mainly a transient buffering mechanism that enables time for later genetic selection. Another concerns stability: whether DTP states represent a distinct biological compartment or a fluid spectrum of stress-adapted phenotypes with variable trajectories and therapeutic relevance. A third concerns translational strategy: whether epigenetic therapies should be positioned mainly as resensitization tools, lineage-state modulators, or broader combinatorial agents. A fourth concerns biomarker interpretation: whether dynamic epigenomic signatures can achieve sufficient analytical robustness and clinical specificity to support repeated use across studies, treatment settings, and platforms (Russo et al., 2024; Sharma et al., 2010; Song et al., 2025; Wang et al., 2025).

### 6.4 What should come next

Future work should prioritize longitudinal, single-cell, and multimodal designs capable of resolving chromatin state, transcriptional circuitry, and lineage trajectories during treatment exposure. Greater mechanistic resolution is needed to distinguish chromatin changes that are functionally required for persistence from those that merely accompany adaptive survival. Biomarker development should focus on fit-for-purpose assays, serial sampling strategies, reproducible implementation, and clinically interpretable endpoints that better reflect the sequential nature of chemoresistance. Real-world studies should be used to extend, not replace, mechanistic and prospective investigation, and should be anchored in target-trial logic, transparent provenance, and bias-aware analysis (ICH, 2024; FDA, 2024).

Overall, the field has moved decisively toward a model in which epigenetic plasticity is central to tumor adaptation under therapy. The challenge is no longer to establish that non-genetic resistance exists, but to define when it matters most, how it becomes stabilized, and which aspects of it can be measured and therapeutically exploited with sufficient rigor to improve patient outcomes.

## References

[B1] Al E. A. Marzese D. M. Menon D. R. Stark M. S. Torrano J. Hammerlindl H. (2018). Distinct Histone Modifications Denote Early stress-induced Drug Tolerance in Cancer, 9. 10.18632/oncotarget.23654 PMC5823586 29492189

[B2] Alexandrova E. Salvati A. Pecoraro G. Lamberti J. Melone V. Sellitto A. (2022). Histone methyltransferase DOT1L as a promising epigenetic target for treatment of solid tumors. Front. Genet. 13, 864612. 10.3389/fgene.2022.864612 35495127 PMC9043692

[B3] Anvisa (2023). Guide No. 64/2023 (Version 1): Good Practice Guide for Real-World Data Studies. Brasília, Brazil: Agência Nacional de Vigilância Sanitária (Anvisa).

[B4] Athieniti E. Spyrou G. M. (2023). A guide to multi-omics data collection and integration for translational medicine. Comput. Struct. Biotechnol. J. 21, 134–149. 10.1016/j.csbj.2022.11.050 36544480 PMC9747357

[B5] Barkley D. Rao A. Pour M. França G. S. Yanai I. (2021). Cancer cell states and emergent properties of the dynamic tumor system. Genome Res. 31, 1719–1727. 10.1101/gr.275308.121 34599005 PMC8494223

[B6] Benchimol E. I. Smeeth L. Guttmann A. Harron K. Moher D. Peteresen I. (2015). The REporting of studies conducted using observational Routinely-collected health data (RECORD) statement. PLoS Med. 12, e1001885. 10.1371/journal.pmed.1001885 26440803 PMC4595218

[B7] Berdasco M. Esteller M. (2019). Clinical epigenetics: seizing opportunities for translation. Nat. Rev. Genet. 20, 109–127. 10.1038/s41576-018-0074-2 30479381

[B8] Bogan S. N. Yi S. V. (2024). Potential role of DNA methylation as a driver of plastic responses to the environment across cells, organisms, and populations. Genome Biol. Evol. 16, evae022. 10.1093/gbe/evae022 38324384 PMC10899001

[B9] Burns L. Kalesnik-Orszulak R. Spring R. Zeegers F. Rutstein M. Hukkelhoven M. (2022). Real world-evidence for regulatory use decision aid: an interactive tool to inform clinical development and regulatory strategies. Adv. Ther. 39, 4772–4778. 10.1007/s12325-022-02257-4 35972721 PMC9379219

[B10] Cashin A. G. Hansford H. J. Hernán M. A. Swanson S. A. Lee H. Jones M. D. (2025). Transparent reporting of observational studies emulating a target trial: the TARGET statement. BMJ 390, e087179. 10.1136/bmj-2025-087179 40903028

[B11] Cazaly E. Saad J. Wang W. Heckman C. Ollikainen M. Tang J. (2019). Making sense of the epigenome using data integration approaches. Front. Pharmacol. 10, 126. 10.3389/fphar.2019.00126 30837884 PMC6390500

[B12] Cézar de Aquino N. Reichert C. O. Alberto de Padua Covas Lage L. Culler H. F. Rosa Roitberg F. S. Rocha V. (2026). Advancing real-world evidence in Brazil: regulatory gaps and global lessons. Lancet Regional Health - Am. 55, 101344. 10.1016/j.lana.2025.101344 PMC12795646 41531545

[B13] Chan J. E. Pan C.-H. Rub J. Guzman G. Krause K. Brown E. (2026). Critical role for a high-plasticity cell state in lung cancer. Nature 651, 231–241. 10.1038/s41586-025-09985-x 41565826 PMC12960256

[B14] Chen Y. Liang R. Li Y. Jiang L. Ma D. Luo Q. (2024). Chromatin accessibility: biological functions, molecular mechanisms and therapeutic application. Signal Transduct. Target Ther. 9, 340. 10.1038/s41392-024-02030-9 39627201 PMC11615378

[B15] Ciriello G. Magnani L. Aitken S. J. Akkari L. Behjati S. Hanahan D. (2024). Cancer evolution: a multifaceted affair. Cancer Discov. 14, 36–48. 10.1158/2159-8290.CD-23-0530 38047596 PMC10784746

[B16] Connolly R. M. Li H. Jankowitz R. C. Zhang Z. Rudek M. A. Jeter S. C. (2017). Combination epigenetic therapy in advanced breast cancer with 5-azacitidine and entinostat: a phase II national cancer institute/stand up to cancer study. Clin. Cancer Res. 23, 2691–2701. 10.1158/1078-0432.CCR-16-1729 27979916 PMC5457329

[B17] Derman B. A. Belli A. J. Battiwalla M. Hamadani M. Kansagra A. Lazarus H. M. (2022). Reality check: real-World evidence to support therapeutic development in hematologic malignancies. Blood Rev. 53, 100913. 10.1016/j.blre.2021.100913 35272867

[B18] Dang A. (2023). Real-world evidence: a primer. Pharm. Med. 37, 25–36. 10.1007/s40290-022-00456-6 PMC9815890 36604368

[B19] Dawson M. A. Kouzarides T. (2012). Cancer epigenetics: from mechanism to therapy. Cell 150, 12–27. 10.1016/j.cell.2012.06.013 22770212

[B20] de Pádua Covas Lage L. A. Culler H. F. Barreto G. C. Reichert C. O. Levy D. de Oliveira Costa R. (2022). Tumor mutation burden involving epigenetic regulatory genes and the RhoA GTPase predicts overall survival in nodal mature T-cell lymphomas. Clin. Epigenetics 14, 180. 10.1186/s13148-022-01395-4 36536430 PMC9764541

[B21] Desai R. K. Lee G. Chatterjee S. Zhou E. J. Yeung C. Y. L. Tadrous M. (2026). Scoping review: investigating target trial emulation approaches in oncology research. Cancer Epidemiol. 102, 103016. 10.1016/j.canep.2026.103016 41762536

[B22] Duan X. Xing Z. Qiao L. Qin S. Zhao X. Gong Y. (2024). The role of histone post-translational modifications in cancer and cancer immunity: functions, mechanisms and therapeutic implications. Front. Immunol. 15, 1495221. 10.3389/fimmu.2024.1495221 39620228 PMC11604627

[B23] Eckersley-Maslin M. A. (2020). Keeping your options open: insights from Dppa2/4 into how epigenetic priming factors promote cell plasticity. Biochem. Soc. Trans. 48, 2891–2902. 10.1042/BST20200873 33336687 PMC7752079

[B24] Elazezy M. Prieske K. Kluwe L. Oliveira-Ferrer L. Peine S. Müller V. (2021). BRCA1 promoter hypermethylation on circulating tumor DNA correlates with improved survival of patients with ovarian cancer. Mol. Oncol. 15, 3615–3625. 10.1002/1878-0261.13108 34601813 PMC8637552

[B25] EMA (2021). Guideline on Registry-based Studies.

[B26] EMA (2025). Real-World Evidence Framework to Support EU Regulatory decision-making: 3Rd Report on the Experience Gained with regulator-led Studies from February 2024 to February 2025.

[B27] ENCePP (2023). Guide on Methodological Standards in Pharmacoepidemiology - Revision 11.

[B28] Eustermann S. Patel A. B. Hopfner K. P. He Y. Korber P. (2024). Energy-driven genome regulation by ATP-Dependent chromatin remodellers. Nat. Rev. Mol. Cell Biol. 25, 309–332. 10.1038/s41580-023-00683-y 38081975 PMC12303036

[B29] Fagnocchi L. Poli V. Zippo A. (2018). Enhancer reprogramming in tumor progression: a new route towards cancer cell plasticity. Cell. Mol. Life Sci. 75, 2537–2555. 10.1007/s00018-018-2820-1 29691590 PMC11105402

[B30] FDA (2011). Guidance for Industry E16 Biomarkers Related to Drug or Biotechnology Product Development: Context, Structure, and Format of Qualification Submissions. 21834216

[B31] FDA (2018). Framework for Fda’s Real-World Evidence Program.

[B32] FDA (2023a). Considerations for the Design and Conduct of Externally Controlled Trials for Drug and Biological Products Guidance for Industry DRAFT GUIDANCE.

[B33] FDA (2023b). Considerations for the Use of Real-World Data and Real-World Evidence to Support Regulatory Decision-Making for Drug and Biological Products Guidance for Industry.

[B34] FDA (2023c). Data standards for drug and biological product submissions containing real-world data. Guid. Industry.

[B35] FDA (2024). Real-World Data: Assessing Electronic Health Records and Medical Claims Data to Support Regulatory Decision-Making for Drug and Biological Products. 10.1002/pds.5444 PMC9320939 35471704

[B36] Flavahan W. A. Gaskell E. Bernstein B. E. (1979). Epigenetic plasticity and the hallmarks of cancer. Science 2017, 357. 10.1126/science.aal2380 28729483 PMC5940341

[B37] França G. S. Baron M. King B. R. Bossowski J. P. Bjornberg A. Pour M. (2024). Cellular adaptation to cancer therapy along a resistance continuum. Nature 631, 876–883. 10.1038/s41586-024-07690-9 38987605 PMC11925205

[B38] Franceschini G. M. Quaini O. Mizuno K. Orlando F. Ciani Y. Ku S. Y. (2024). Noninvasive detection of neuroendocrine prostate cancer through targeted cell-free DNA methylation. Cancer Discov. 14, 424–445. 10.1158/2159-8290.CD-23-0754 38197680 PMC10905672

[B39] Galluzzi L. Kroemer G. (2022). Immuno-epigenetic escape of cancer stem cells. Nat. Immunol. 23, 1300–1302. 10.1038/s41590-022-01293-0 36002649

[B40] Gatla H. R. Zou Y. Uddin M. M. Singha B. Bu P. Vancura A. (2017). Histone deacetylase (HDAC) inhibition induces IκB kinase (IKK)-Dependent interleukin-8/CXCL8 expression in ovarian cancer cells. J. Biol. Chem. 292, 5043–5054. 10.1074/jbc.M116.771014 28167529 PMC5377816

[B41] Gatto N. M. Vititoe S. E. Rubinstein E. Reynolds R. F. Campbell U. B. (2023). A structured process to identify fit-for-purpose study design and data to generate valid and transparent real-world evidence for regulatory uses. Clin. Pharmacol. Ther. 113, 1235–1239. 10.1002/cpt.2883 36871138

[B42] Ghoderao P. Kwiatkowska-Borowczyk E. Sahare S. Dams-Kozlowska H. (2025). Targeting drug-tolerant persister cancer cells: can nanomaterial-based strategies be helpful for Anti-DTP therapies? Pharmaceutics 17, 1428. 10.3390/pharmaceutics17111428 41304766 PMC12655282

[B43] Glasspool R. M. Brown R. Gore M. E. Rustin G. J. S. McNeish I. A. Wilson R. H. (2014). A randomised, phase II trial of the DNA-Hypomethylating agent 5-aza-2′-deoxycytidine (decitabine) in combination with carboplatin vs carboplatin alone in patients with recurrent, partially platinum-sensitive ovarian cancer. Br. J. Cancer 110, 1923–1929. 10.1038/bjc.2014.116 24642620 PMC3992493

[B44] Goyal Y. Busch G. T. Pillai M. Li J. Boe R. H. Grody E. I. (2023). Diverse clonal fates emerge upon drug treatment of homogeneous cancer cells. Nature 620, 651–659. 10.1038/s41586-023-06342-8 37468627 PMC10628994

[B45] Guo L. Lee Y. T. Zhou Y. Huang Y. (2022). Targeting epigenetic regulatory machinery to overcome cancer therapy resistance. Semin. Cancer Biol. 83, 487–502. 10.1016/j.semcancer.2020.12.022 33421619 PMC8257754

[B46] Hangauer M. J. Viswanathan V. S. Ryan M. J. Bole D. Eaton J. K. Matov A. (2017). Drug-tolerant persister cancer cells are vulnerable to GPX4 inhibition. Nature 551, 247–250. 10.1038/nature24297 29088702 PMC5933935

[B47] Hegi M. E. Diserens A.-C. Gorlia T. Hamou M.-F. de Tribolet N. Weller M. (2005). MGMT gene silencing and benefit from temozolomide in glioblastoma. N. Engl. J. Med. 352, 997–1003. 10.1056/NEJMoa043331 15758010

[B48] Hermanova I. Zúñiga-García P. Caro-Maldonado A. Fernandez-Ruiz S. Salvador F. Martín-Martín N. (2020). Genetic manipulation of LKB1 elicits lethal metastatic prostate cancer. J. Exp. Med. 217, e20191787. 10.1084/jem.20191787 32219437 PMC7971141

[B49] Hernán M. A. Robins J. M. (2016). Using big data to emulate a target trial when a randomized trial is not available. Am. J. Epidemiol. 183, 758–764. 10.1093/aje/kwv254 26994063 PMC4832051

[B50] Hiramatsu K. Barrett A. Miyata Y. (2021). Current status, challenges, and future perspectives of real-world data and real-world evidence in Japan. Drugs Real World Outcomes 8, 459–480. 10.1007/s40801-021-00266-3 34148219 PMC8605941

[B51] Huang H. Y. Wang L. Xu S. Jia S. P. Kong D. D. Zhang X. J. (2025). Target trial emulation in oncology: current use and future directions. Mil. Med. Res. 12, 99. 10.1186/s40779-026-00685-9 41810148 PMC12930863

[B52] ICH (2021). General Considerations for Clinical Studies (E8(R1)).

[B53] ICH (2000). Choice of Control Group and Related Issues in Clinical Trials (E10). 12356096

[B54] ICH (2005). Pharmacovigilance Planning (E2E).

[B55] ICH (2019). Addendum on Estimands and Sensitivity Analysis in Clinical Trials to the Guideline on Statistical Principles for Clinical Trials. E9(R1)).

[B56] ICH (2024). ICH M14 Guideline on General Principles on Planning, Designing, Analysing, and Reporting of Non-interventional Studies that Utilise Real-World Data for Safety Assessment of Medicines.

[B57] ICH (2025). GUIDELINE FOR GOOD CLINICAL PRACTICE E6(R3).

[B58] ICH. Definitions for Genomic Biomarkers (2007). Pharmacogenomics, Pharmacogenetics, Genomic Data Sample Coding Categ. (E15).

[B59] Joun G. L. Kempe E. G. Chen B. Sterling J. R. Abbassi R. H. Friess D. (2025). Histone methyltransferase PRDM9 promotes survival of drug-tolerant persister cells in glioblastoma. Nat. Commun. 16, 10905. 10.1038/s41467-025-65888-5 41397959 PMC12705669

[B60] Juergens R. A. Wrangle J. Vendetti F. P. Murphy S. C. Zhao M. Coleman B. (2011). Combination epigenetic therapy has efficacy in patients with refractory advanced non-small cell lung cancer. Cancer Discov. 1, 598–607. 10.1158/2159-8290.CD-11-0214 22586682 PMC3353724

[B61] Kawaf R. R. Ramadan W. S. El-Awady R. (2024). Deciphering the interplay of histone post-translational modifications in cancer: co-Targeting histone modulators for precision therapy. Life Sci. 346, 122639. 10.1016/j.lfs.2024.122639 38615747

[B62] Klemm S. L. Shipony Z. Greenleaf W. J. (2019). Chromatin accessibility and the regulatory epigenome. Nat. Rev. Genet. 20, 207–220. 10.1038/s41576-018-0089-8 30675018

[B63] Kondrashova O. Topp M. Nesic K. Lieschke E. Ho G. Y. Harrell M. I. (2018). Methylation of all BRCA1 copies predicts response to the PARP inhibitor rucaparib in ovarian carcinoma. Nat. Commun. 9, 3970. 10.1038/s41467-018-05564-z 30266954 PMC6162272

[B64] Krause J. H. Saver R. S. (2018). Real-world evidence in the real world: beyond the FDA. Am. J. Law Med. 44, 161–179. 10.1177/0098858818789423 30106647

[B65] Lage L. A. de P. C. Culler H. F. Reichert C. O. da Siqueira S. A. C. Pereira J. (2023). Angioimmunoblastic T-cell lymphoma and correlated neoplasms with T-cell follicular helper phenotype: from molecular mechanisms to therapeutic advances. Front. Oncol. 13, 1177590. 10.3389/fonc.2023.1177590 37182145 PMC10169672

[B66] Lalla M. Ratnani A. Yang J. Wang M. Cheng H. (2026). Drug-tolerant persister cells and tumor dormancy in NSCLC: a new frontier in overcoming therapeutic resistance. Cancers (Basel) 18, 779. 10.3390/cancers18050779 41827714 PMC12984249

[B67] Lau C. Y. Jamali F. Loebenberg R. Lau C. (2022). Health Canada Usage of Real World Evidence (RWE) in Regulatory Decision Making Compared with FDA/EMA Usage Based on Publicly Available Information, 25. 10.18433/jpps32715 35760071

[B68] Levy D. Ferreira MCMR Reichert C. O. de Almeida L. V. Brocardo G. Lage LAPC (2020). Cell cycle changes, DNA ploidy, and PTTG1 gene expression in HTLV-1 patients. Front. Microbiol. 11, 1778. 10.3389/fmicb.2020.01778 32793179 PMC7393187

[B69] Li D. Shu X. Zhu P. Pei D. (2021). Chromatin accessibility dynamics during cell fate reprogramming. EMBO Rep. 22, e51644. 10.15252/embr.202051644 33480184 PMC7857421

[B70] Li X. Gardner E. E. Molina-Pinelo S. Wilhelm C. Mu P. Quintanal-Villalonga Á. (2025). Lineage plasticity and histological transformation: tumor histology as a spectrum. Cell Res. 35, 803–823. 10.1038/s41422-025-01180-x 41023204 PMC12589604

[B71] Li J. J. Vasciaveo A. Karagiannis D. Sun Z. Gretarsson K. H. Chen X. (2026). NSD2 targeting reverses plasticity and drug resistance in prostate cancer. Nature 649, 216–226. 10.1038/s41586-025-09727-z 41299174 PMC12727498

[B72] Liang Y. Cao H. Tang Z. Li S. Yang G. Dong S. (2025). The role of LKB1 in prostate cancer: implications for tumor progression and therapy. Front. Cell Dev. Biol. 13, 1629844. 10.3389/fcell.2025.1629844 40874022 PMC12378237

[B73] Liu S. Jiang A. Tang F. Duan M. Li B. (2025a). Drug-induced tolerant persisters in tumor: mechanism, vulnerability and perspective implication for clinical treatment. Mol. Cancer 24, 150. 10.1186/s12943-025-02323-9 40413503 PMC12102949

[B74] Liu F. He L. Yu M. Chen J. Huang Y. Ma W. (2025b). Histone modification networks reshape the metabolism and treatment landscape of urological cancers. Front. Biosci. Landmark Ed. 30, 42831. 10.31083/FBL42831 41351419

[B75] Lord S. J. Horvath A. R. Sandberg S. Monaghan P. J. M. Cobbaert C. Reim M. (2025). Is this test fit-for-purpose? Principles and a checklist for evaluating the clinical performance of a test in the new era of *in vitro* diagnostic (IVD) regulation. Crit. Rev. Clin. Lab. Sci. 62, 182–197. 10.1080/10408363.2025.2453148 39912349

[B76] Malone H. A. Roberts C. W. M. (2024). Chromatin remodellers as therapeutic targets. Nat. Rev. Drug Discov. 23, 661–681. 10.1038/s41573-024-00978-5 39014081 PMC11534152

[B77] Matei D. Fang F. Shen C. Schilder J. Arnold A. Zeng Y. (2012). Epigenetic resensitization to platinum in ovarian cancer. Cancer Res. 72, 2197–2205. 10.1158/0008-5472.CAN-11-3909 22549947 PMC3700422

[B78] Mehta A. Stanger B. Z. (2024). Lineage plasticity: the new cancer hallmark on the block. Cancer Res. 84, 184–191. 10.1158/0008-5472.CAN-23-1067 37963209 PMC10841583

[B79] Moore P. C. Henderson K. W. Classon M. (2023). The epigenome and the many facets of cancer drug tolerance. Adv. Cancer Res. 158, 1–39. 10.1016/bs.acr.2022.12.002 36990531

[B80] Musella M. Guarracino A. Manduca N. Galassi C. Ruggiero E. Potenza A. (2022). Type I IFNs promote cancer cell stemness by triggering the epigenetic regulator KDM1B. Nat. Immunol. 23, 1379–1392. 10.1038/s41590-022-01290-3 36002648 PMC9477743

[B81] Nianogo R. A. Benmarhnia T. O’Neill S. (2023). A comparison of quasi-experimental methods with data before and after an intervention: an introduction for epidemiologists and a simulation study. Int. J. Epidemiol. 52, 1522–1533. 10.1093/ije/dyad032 37023467 PMC10555819

[B82] Palma F. R. Coelho D. R. Pulakanti K. Sakiyama M. J. Huang Y. Ogata F. T. (2024). Histone H3.1 is a chromatin-embedded redox sensor triggered by tumor cells developing adaptive phenotypic plasticity and multidrug resistance. Cell Rep. 43, 113897. 10.1016/j.celrep.2024.113897 38493478 PMC11209755

[B83] Perlman B. S. Burget N. Zhou Y. Schwartz G. W. Petrovic J. Modrusan Z. (2024). Enhancer-promoter hubs organize transcriptional networks promoting oncogenesis and drug resistance. Nat. Commun. 15, 8070. 10.1038/s41467-024-52375-6 39277592 PMC11401928

[B84] Pradhan S. Kabekkodu S. P. Eswaran S. Kumar N. A. N. Upadhya D. Chakrabarty S. (2026). Inflammatory milieu and role of epigenetic modifications in high-grade serous ovarian cancer. Clin. Epigenetics 18, 47. 10.1186/s13148-026-02080-6 41680927 PMC12997951

[B85] Pu Y. Li L. Peng H. Liu L. Heymann D. Robert C. (2023). Drug-tolerant persister cells in cancer: the cutting edges and future directions. Nat. Rev. Clin. Oncol. 20, 799–813. 10.1038/s41571-023-00815-5 37749382

[B86] Purpura C. A. Garry E. M. Honig N. Case A. Rassen J. A. (2022). The role of real-world evidence in FDA-approved new drug and biologics license applications. Clin. Pharmacol. Ther. 111, 135–144. 10.1002/cpt.2474 34726771 PMC9299054

[B87] Ramirez M. Rajaram S. Steininger R. J. Osipchuk D. Roth M. A. Morinishi L. S. (2016). Diverse drug-resistance mechanisms can emerge from drug-tolerant cancer persister cells. Nat. Commun. 7, 10690. 10.1038/ncomms10690 26891683 PMC4762880

[B88] Russo M. Chen M. Mariella E. Peng H. Rehman S. K. Sancho E. (2024). Cancer drug-tolerant persister cells: from biological questions to clinical opportunities. Nat. Rev. Cancer 24, 694–717. 10.1038/s41568-024-00737-z 39223250 PMC12622869

[B89] Seidel E. von Karstedt S. (2022). Extrinsic cell death pathway plasticity: a driver of clonal evolution in cancer? Cell Death Discov. 8, 465. 10.1038/s41420-022-01251-7 36435845 PMC9701215

[B90] Semaan K. Nawfal R. Canniff J. Savignano H. Seo J. H. Siegmund S. E. (2025). Plasma epigenomic profiling reveals treatment-emergent squamous transformation in prostate cancer. NPJ Precis. Oncol. 9, 233. 10.1038/s41698-025-01031-3 40634667 PMC12241565

[B91] Shaffer S. M. Dunagin M. C. Torborg S. R. Torre E. A. Emert B. Krepler C. (2017). Rare cell variability and drug-induced reprogramming as a mode of cancer drug resistance. Nature 546, 431–435. 10.1038/nature22794 28607484 PMC5542814

[B92] Sharma S. V. Lee D. Y. Li B. Quinlan M. P. Takahashi F. Maheswaran S. (2010). A chromatin-mediated reversible drug-tolerant state in cancer cell subpopulations. Cell 141, 69–80. 10.1016/j.cell.2010.02.027 20371346 PMC2851638

[B93] Sherman R. E. Anderson S. A. Dal Pan G. J. Gray G. W. Gross T. Hunter N. L. (2016). Real-world Evidence—What is it and what can it tell Us? N. Engl. J. Med. 375, 2293–2297. 10.1056/NEJMsb1609216 27959688

[B94] Sipola J. Munzur A. D. Kwan E. M. Seo C. C. Y. Hauk B. J. Parekh K. (2025). Plasma cell–free DNA chromatin immunoprecipitation profiling depicts phenotypic and clinical heterogeneity in advanced prostate cancer. Cancer Res. 85, 791–807. 10.1158/0008-5472.CAN-24-2052 39652574 PMC11832346

[B95] Song J. Yang P. Chen C. Ding W. Tillement O. Bai H. (2025). Targeting epigenetic regulators as a promising avenue to overcome cancer therapy resistance. Signal Transduct. Target Ther. 10, 219. 10.1038/s41392-025-02266-z 40675967 PMC12271501

[B96] Soragni A. Knudsen E. S. O’Connor T. N. Tognon C. E. Tyner J. W. Gini B. (2025). Acquired resistance in cancer: towards targeted therapeutic strategies. Nat. Rev. Cancer 25, 613–633. 10.1038/s41568-025-00824-9 40461793 PMC12307123

[B97] Strauss J. Figg W. D. (2015). Epigenetic approaches to overcoming chemotherapy resistance. Lancet Oncol. 16, 1013–1015. 10.1016/S1470-2045(15)00231-4 26296953 PMC6387683

[B98] Sundaram L. Kumar A. Zatzman M. Salcedo A. Ravindra N. Shams S. (1979). Single-cell chromatin accessibility reveals malignant regulatory programs in primary human cancers. Science 2024, 385. 10.1126/science.adk9217 PMC12289346 39236169

[B99] Tan G. S. Q. Sloan E. K. Lambert P. Kirkpatrick C. M. J. Ilomäki J. (2022). Drug repurposing using real-world data. Drug Discov. Today 28, 103422. 10.1016/j.drudis.2022.103422 36341896

[B100] Tao L. Zhou Y. Luo Y. Qiu J. Xiao Y. Zou J. (2024). Epigenetic regulation in cancer therapy: from mechanisms to clinical advances. MedComm - Oncol. 3, e59. 10.1002/mog2.59

[B101] Teneng I. Picchi M. A. Leng S. Dagucon C. P. Ramalingam S. Tellez C. S. (2019). DNA-PKc deficiency drives pre-malignant transformation by reducing DNA repair capacity in concert with reprogramming the epigenome in human bronchial epithelial cells. DNA Repair (Amst) 79, 1–9. 10.1016/j.dnarep.2019.04.006 31055244 PMC6551272

[B102] Timbie J. W. Kim A. Y. Concannon T. W. (2021). Health policy analysis use of real-world evidence for regulatory approval and coverage of medical devices: a landscape assessment. VALUE HEALTH 24, 1792–1798. 10.1016/j.jval.2021.07.003 34838277

[B103] Wallach J. D. Deng Y. Polley E. C. Dhruva S. S. Herrin J. Quinto K. (2023). Assessing the use of observational methods and real-world data to emulate ongoing randomized controlled trials. Clin. Trials 20, 689–698. 10.1177/17407745231193137 37589143 PMC10843567

[B104] Wang S. V. Pinheiro S. Hua W. Arlett P. Uyama Y. Berlin J. A. (2021). STaRT-RWE: structured template for planning and reporting on the implementation of real world evidence studies. BMJ 372, m4856. 10.1136/bmj.m4856 33436424 PMC8489282

[B105] Wang N. Ma T. Yu B. (2023a). Targeting epigenetic regulators to overcome drug resistance in cancers. Signal Transduct. Target Ther. 8, 69. 10.1038/s41392-023-01341-7 36797239 PMC9935618

[B106] Wang S. V. Pottegård A. Crown W. Arlett P. Ashcroft D. M. Benchimol E. I. (2023b). HARmonized protocol template to enhance reproducibility of hypothesis evaluating real-world evidence studies on treatment effects: a good practices report of a joint ISPE/ISPOR task force. Pharmacoepidemiol Drug Saf. 32, 44–55. 10.1002/pds.5507 36215113 PMC9771861

[B107] Wang Z. Townley S. L. Zhang S. Liu M. Li M. Labaf M. (2024). FOXA2 rewires AP-1 for transcriptional reprogramming and lineage plasticity in prostate cancer. Nat. Commun. 15, 4914. 10.1038/s41467-024-49234-9 38851846 PMC11162502

[B108] Wang Z. Wang M. Dong B. Wang Y. Ding Z. Shen S. (2025). Drug-tolerant persister cells in cancer: bridging the gaps between bench and bedside. Nat. Commun. 16, 10048. 10.1038/s41467-025-66376-6 41249194 PMC12623778

[B109] Xiang R. R. Lee S. A. Tyndall C. F. Bhatia A. R. Yin J. J. Singler C. (2025). CRISPR screening identifies regulators of enhancer-mediated androgen receptor transcription in advanced prostate cancer. Cell Rep. 44, 115312. 10.1016/j.celrep.2025.115312 39954255 PMC11867844

[B110] Zboril E. Yoo H. Chen L. Liu Z. (2021). Dynamic interactions of transcription factors and enhancer reprogramming in cancer progression. Front. Oncol. 11, 753051. 10.3389/fonc.2021.753051 34616687 PMC8488287

[B111] Zhang L. Goodrich D. W. (2022). RB1, cancer lineage plasticity, and therapeutic resistance. Annu. Rev. Cancer Biol. Annu. Rev Cancer Biol 6, 201–222. 10.1146/annurev-cancerbio-070120

[B112] Zhu Y. Pittella-Silva F. Wang Y. Wang T. Long F. (2023). Editorial: epigenetic regulation and therapy resistance in cancer. Front. Pharmacol. 13, 1119073. 10.3389/fphar.2022.1119073 36699076 PMC9868549

[B113] Zippo A. Beyes S. (2025). Molecular mechanisms altering cell identity in cancer. Oncogene 44, 2117–2126. 10.1038/s41388-025-03314-2 40011573

